# Hypoxia Upregulates RIPK1 via the HIF‐1α–JAK–STAT Pathway Leading to Astrocyte Necroptosis to Promote Cavitation After Spinal Cord Injury

**DOI:** 10.1002/cns.70895

**Published:** 2026-05-11

**Authors:** Hangchuan Bi, Wan Zhang, Hao Duan, Chao Wang, Xianglin Shen, Gang Jiang, Haiyan Xue, Rongji Yan, Yuan Xu, Yihe Zhang, Haoyu Zhao, Fei He, Zhihua Wang

**Affiliations:** ^1^ Trauma Center, the First Affiliated Hospital of Kunming Medical University Kunming China; ^2^ Kunming Medical University Affiliated Qujing Hospital Qujing China

**Keywords:** cavity of injury, HIFα, hypoxic, JAK/STAT, necroptosis, spinal cord injury

## Abstract

**Background:**

The pathological mechanisms after spinal cord injury (SCI) are complex, making SCI repair a major clinical challenge for clinical treatment. Currently, there is no effective method to effectively improve the prognosis of patients. A deeper understanding of post‐SCI pathology may reveal new targets and strategies for clinical intervention.

**Methods:**

A rat SCI model was constructed, and HE staining, immunohistochemistry, and tissue immunofluorescence double staining were used to detect the pathological changes and cell death in injured tissue. Human astrocytes were subjected to hypoxic conditions, and necroptosis and apoptosis mechanisms were investigated using CCK8, immunofluorescence, Western blot, transmission electron microscopy, and related assays.

**Results:**

In vivo animal experiments demonstrated that after SCI, rats exhibited the formation of damage cavities and increased astrocyte apoptosis in the lesion area, along with significantly elevated expression of key proteins in the HIF‐1α pathway and necroptosis. In vitro cellular experiments showed that hypoxia induced the elevation of the expression of key factors of human astrocytes HIF1α and JAK/STAT, necroptosis, and the conversion of the cells to an A1‐type phenotype with the use of the necroptosis inhibitors, JAK/STAT inhibitors or knockdown of HIF1α or knockdown of RIP1 expression could reverse the hypoxia‐induced effects.

**Conclusion:**

In conclusion, hypoxia promotes COI formation after SCI by activating the HIF‐1α–JAK–STAT pathway, which in turn leads to necroptosis of astrocytes.

## Graphical abstract

This study established a rat spinal cord injury (SCI) model and an in vitro hypoxic astrocyte model, confirming that hypoxia promotes the formation of the cavity of injury (COI) after SCI by activating the HIF‐1α/JAK/STAT pathway, which in turn induces astrocytic necroptosis and ultimately drives the pathological progression of SCI.

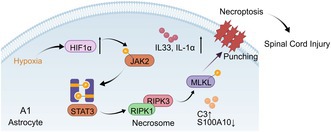

## 1 Introduction

Spinal cord injury (SCI) refers to structural and functional damage caused by events such as traffic accidents, falls, and sports injuries, which in turn leads to below‐plane sensory, motor, and autonomic dysfunction [1, 2]. In recent years, the prevalence of SCI has been on the rise due to population aging and the growing use of motor vehicles. Patients with SCI are often at risk for secondary diseases. This results in a serious social and economic burden for their families and significantly increases the probability of premature death [3]. Currently, the management of SCI focuses on surgery, medication, and rehabilitation. However, the limitations of these treatments and the complexity of the pathomechanisms of SCI mean that improving patient prognosis remains a major challenge. Consequently, in‐depth exploration of the mechanisms of SCI may lead to the development of potential targets and more effective strategies for its clinical treatment.

Sudden axonal severance and subsequent neuronal death caused by SCI usually result in irreversible neurological deficits. The pathological process of SCI is complex and involves both primary and secondary mechanisms of injury, with the primary injury caused by external forces typically being irreversible. Therefore, timely and effective interventions for secondary SCI injury (SSCI), aimed at promoting neuronal regeneration and recovery, are the focus of the current therapeutic strategies [4, 5]. SSCI is a slow‐onset, dynamic pathological process that occurs from min to years after SCI, encompassing spinal cord edema, ischemia and hypoxia, inflammation, oxidative stress, and astrocyte proliferation. These changes lead to the formation of a fibro‐glial cell scar at the site of injury and a permanent, edematous fluid‐filled cavity of injury (COI) [6, 7]. The COI is a reservoir of edematous fluid within the tissues surrounding the central nervous system (CNS), and its fluid milieu is a major contributor to the persistence of inflammation and thus the promotion of SSCI [8]. Astrocytes, as the main functional cells in the CNS, provide metabolic and structural support to neurons [9]. Reactive astrocytes are the main component of glial scarring; however, their proliferation and associated inflammation promote degeneration and death of neurons, oligodendrocytes and neural progenitor cells [5, 10]. Studies have shown that microglial activation can induce A1‐type astrocyte activation through secretion of pro‐inflammatory cytokines such as IL‐1α and TNF, which in turn promote neuronal death [11]. Another report also showed that type A1 astrocytes were activated in mice with advanced SCI, thereby promoting inflammation, inducing neuronal apoptosis, and inhibiting axon growth [12]. In addition, genes related to neuroprotection and repair were significantly upregulated in A2‐type astrocytes in response to hypoxia induction [13]. Collectively, these studies demonstrated some mechanisms underlying the different phenotypes of astrocytes in SCI progression and neuroprotection, but their role in COI formation after SCI under hypoxic or inflammatory environments is not clear.

Studies have shown that SCI progression is directly related to diverse forms of programmed cell death (PCD), including apoptosis, necroptosis, and autophagy in neuronal cells [14]. It has been reported that COI formation and progressive enlargement after SCI in mice are always surrounded by reactive astrocytes and that astrocyte loss is associated with RIP3/MLKL‐mediated necroptosis rather than apoptosis and autophagy [10]. Accumulating evidence demonstrates that necroptosis in SCI is often accompanied by an active inflammatory immune response, and that inflammation is a key determinant of the severity of its injury [15]. Thus, necroptosis‐mediated astrocyte loss around COI may be an important contributor to the progression of SSCI. For instance, RIPK3/MLKL‐mediated necroptosis in ischemia exacerbates neuronal death while promoting the release of pro‐inflammatory factors such as TNF‐α, which further aggravates tissue injury [16]. Moreover, the use of the necroptosis inhibitor Necrostatin‐1 (Nec‐1) enhances the neuroprotective effects of hypothermia in hypoxic Lewis rats [17]. The use of Nec‐1 in C57BL mice enhanced the resistance to hypoxic brain injury [18]. This studies suggest the protective effect of necroptosis inhibitors against brain injury or neurons in hypoxia, while the tissues or cells in the injured area after SCI often suffer from ischemia and hypoxia and other phenomena. However, the mechanism of the role of hypoxia and necroptosis‐mediated biological responses in the formation of COI in injured tissues after SCI has not been studied.

In this study, we constructed an animal model of SCI and an in vitro cell model and performed a series of biological experiments to demonstrate that hypoxia after SCI activates the hypoxia‐inducible factor (HIF‐1α)‐JAK–STAT pathway, which in turn leads to necroptosis of astrocytes and thus promotes the formation of COI post‐SCI. The present study aimed to elucidate the specific mechanism of COI formation after SCI, with the goal of providing new potential targets and effective strategies for SCI treatment.

## 2 Methods

### 2.1 Animals

Twenty healthy male Sprague–Dawley (SD) rats of specific pathogen‐free (SPF) grade, aged 8 weeks and weighing 200–250 g, were obtained from Vital River Laboratory Animal Technology Co. Ltd. (Beijing, China). The rats were housed in a controlled environment maintained at 22°C–26°C with a 12‐h light/12‐h dark cycle. Subsequently, the animals were randomly divided into two groups: a control group and a model group, with 10 rats in each group. All animal experimental protocols involved in this study were reviewed and approved by the Animal Experiment Ethical Committee of Kunming Medical University (Approval No.: kmmu20240627).

### 2.2 SCI Model Establishment and Treatment In Vivo

**FIGURE 1:**
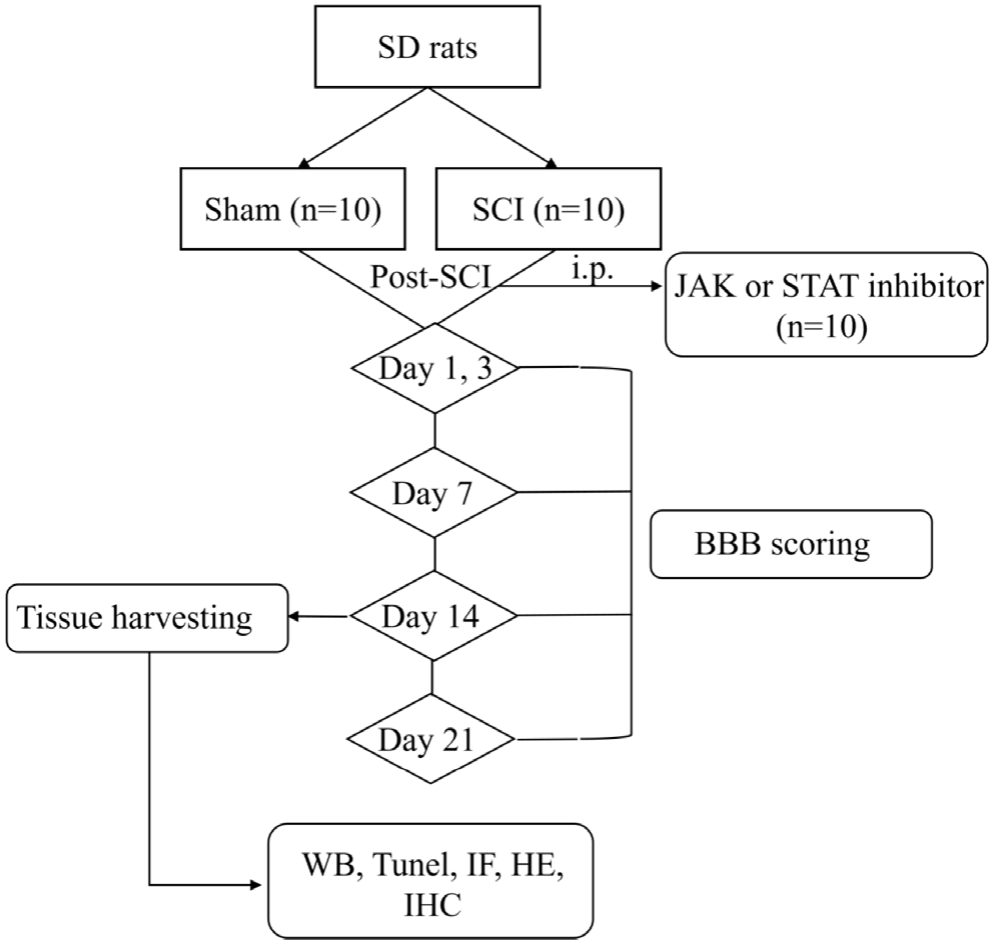
Flowchart of animal experiment.

SD rats were anesthetized by intraperitoneal injection of 1% sodium pentobarbital at a dose of 50 mg/kg, and placed in a prone position. The sham‐operated group only underwent exposure of the spinal cord without additional intervention. For the SCI model group, modeling was performed as follows: Hair in the region centered on the most prominent point of the thoracic spinal curvature was clipped first; the surgical site was then disinfected with iodophor, after which an incision was made in the skin to expose the fat layer on the dorsal aspect of the neck. Next, the fascial layer and muscles were dissected layer by layer to reveal the spinous processes. The T10 and T11 vertebrae were identified based on anatomical landmarks: T11 inclines cephalad, T9 inclines caudad, and T10 remains neutral with a slight posterior inclination. The spinous processes of T10 and T11, as well as laminae of T10–T11, were removed to fully expose the dorsal and bilateral sides of the spinal cord. Contusion injury was induced using the optimized Allen's method [19]: a 10‐g weight was permitted to fall freely from a height of 2.5 cm, impacting the exposed dura and spinal cord (successful model establishment was evidenced by tail twitching and hindlimb paralysis observed post‐modeling). The surgical wound was irrigated with sterile PBS, after which the incised area was sutured sequentially, and antibiotic treatment was administered. After surgery, the JAK inhibitor group and STAT inhibitor group were administered 10 mg/kg of tofacitinib (MCE, Shanghai, China) and stattic (MCE, Shanghai, China), respectively, via intraperitoneal (i.p.) injection three times per week for 3 consecutive weeks. The sham‐operated group and model group received normal saline via the same administration protocol. Post‐modeling, the rats received manual bladder expression 2–3 times daily until they recovered the ability to develop a voluntary micturition reflex. The subsequent experimental time nodes and testing contents are shown in Figure 1.

### 2.3 HE Staining

Tissues were first trimmed into pieces with a thickness not exceeding 0.5 cm, fixed, and then transferred to embedding cassettes. Following a 30‐min rinsing step, the sections were dehydrated with gradient concentrations of alcohol, cleared in xylene, and infiltrated with paraffin. The embedded blocks were sectioned into thin slices using a microtome; these slices were floated on warm water to facilitate mounting onto glass slides and subsequently dried by heating. The sections were deparaffinized in xylene and rehydrated through a descending alcohol gradient. Next, the sections were stained in an aqueous hematoxylin solution for 10 min, briefly differentiated, and then rinsed under running tap water for 1 h for blueing. Following dehydration via gradient alcohol, the sections were counterstained with eosin solution for 2–3 min, after which further dehydration and transparency treatment were performed. Finally, a mounting medium was applied and coverslips were placed to seal the sections, and the prepared samples were observed under a light microscope.

### 2.4 Immunohistochemical Assay

Tissue samples were sectioned, fixed, rinsed, and embedded in paraffin. The prepared sections were immersed in a hydrogen peroxide blocking solution for 10–15 min of incubation, after which they were rinsed twice with buffer (5 min per wash). Ultra V Block was applied, and the sections were incubated at room temperature for 5 min to block non‐specific background signals; followed by two 5‐min buffer rinses. Subsequently, primary antibody (anti‐GFAP) was added dropwise, and incubation was carried out at 37°C for 1–2 h, then rinsed twice with buffer (5 min each). HRP polymer was applied, incubated at room temperature for 30 min, and rinsed twice with buffer (5 min each). Next, a streptavidin–HRP working solution was applied and incubated at 37°C for 10–30 min, after which sections were rinsed three times with PBS (5 min each). Chromogenic reaction was initiated using DAB, with the development process lasting 3–15 min. Finally, the samples were thoroughly rinsed, followed by counterstaining and mounting with coverslips; they were then observed under a microscope.

### 2.5 TUNEL Staining

The TUNEL assay was conducted with the One‐Step TUNEL Apoptosis Detection Kit (Green Fluorescence), which was purchased from Beyotime Biotechnology (Shanghai, China). First, brain tissues were harvested from rats in each group, and paraffin sections were prepared through standard dewaxing and rehydration. The tissue sections were then incubated with a 20 μg/mL protease K solution at 37°C for 15 min, after which they were washed twice with PBS (5 min each). Subsequently, 50 μL of converter‐POD was added, followed by incubation at 37°C for 60 min; post‐incubation, the sections were rinsed three times with PBS, with each wash lasting 5 min. Next, 50 μL of converter‐POD was applied to the sections, and incubation was continued at 37°C for 30 min. After this incubation step, the sections were again washed three times with PBS (5 min each). Finally, the sections were mounted with an anti‐fade medium and observed under a fluorescence microscope—TUNEL‐positive cells were identified by green fluorescence in the nuclei.

### 2.6 Double Immunofluorescence Staining

After fixation, dehydration, embedding and sectioning, the tissues were subjected to heat‐induced antigen retrieval with 0.01 mol/L citrate buffer (pH 6.0). The sections were immersed in the retrieval solution and heated in a 95°C constant‐temperature water bath for 20 min. Following natural cooling to room temperature, the sections were rinsed with PBS three times for 5 min each. The sections were then incubated in PBS supplemented with 0.3% Triton X‐100 for 20 min at room temperature. After removing the permeabilization solution, the sections were blocked with 5% goat serum for 30 min at room temperature. The blocking solution was discarded, and diluted rat anti‐GFAP primary antibody (Abcam, Cambridge, UK) was added; the sections were incubated in a humidified chamber at 4°C overnight. On the next day, the sections were washed with PBST three times for 5 min each, followed by incubation with CY3‐conjugated goat anti‐rat secondary antibody (Abcam, Cambridge, UK) for 1 h at room temperature in the dark, and then rinsed three times with PBST. The sections were blocked again with 5% rabbit serum for 30 min at room temperature, after which rabbit anti‐RIPK3 primary antibody (Abcam, Cambridge, UK) was applied, and incubation was performed in a humidified chamber at 4°C overnight. On the following day, after washing with PBST, the sections were incubated with DyLight 488‐labeled goat anti‐rabbit secondary antibody (Abcam, Cambridge, UK) for 1 h at room temperature in the dark, and then washed thoroughly with PBST three times. DAPI‐containing anti‐fade mounting medium was added to the sections, and a coverslip was placed over the tissue. Gentle pressure was applied to spread the mounting medium evenly, and the edges of the coverslip were sealed with nail polish. The mounted slides were observed and imaged under a Leica TCS‐SP5 laser scanning confocal microscope.

### 2.7 Motor Function Evaluation

Hindlimb motor function recovery was evaluated in rats at 1, 3, 7, 14, and 21 days after SCI. Briefly, rats were placed on a platform, and their hindlimb locomotion and movement were observed and documented. A blinded assessment was performed according to the Basso, Beattie and Bresnahan (BBB) locomotor rating scale, with scores ranging from 0 (complete paralysis) to 21 (normal motor function).

### 2.8 Cell Culture

Human astrocytes were purchased from Pricella Biotechnology (Wuhan, China). Cells in the control group were cultured in an incubator under standard conditions (37°C, 95% relative humidity, 5% CO_2_, and 21% O_2_). To mimic the chronic hypoxic microenvironment of the syrinx in SCI, hypoxia was induced by incubating the cells in a 1% O_2_ atmosphere for 48 h, and this group was designated as the hypoxia group [20]. All cells across groups were cultured in human astrocytes‐specific complete medium (Pricella, China). After 24 h of culture, the cells were treated with different experimental reagents as required: necroptosis inhibitor (Nec‐1), STAT3 inhibitor (SH‐4‐54) or activator (Colivelin), and JAK inhibitor (Ruxolitinib). All these reagents were sourced from MCE (Shanghai, China). Relevant detection assays were subsequently performed on the cells 48 h after the reagent treatment.

### 2.9 Cell Transfection

shRNA targeting HIF1α (sh‐HIF1α) and RIPK1 (sh‐RIPK1) were obtained from Tsingke Biotechnology Co. Ltd. (Beijing, China). Human astrocytes were seeded and cultured until they reached 70%–90% confluence, which is the optimal density for transfection. Lipofectamine 3000 reagent (Invitrogen, USA) was first diluted with serum‐free medium and vigorously mixed to ensure homogeneity. Meanwhile, shRNA was diluted in serum‐free medium separately to prepare a premix; subsequently, the previously diluted Lipofectamine 3000 reagent was added to this shRNA premix, followed by thorough mixing. The two prepared solutions were then combined, mixed well again, and incubated at room temperature for 10–15 min to allow the formation of transfection complexes. Finally, the transfection mixture was added to the cell culture medium in the culture wells, and the medium was gently agitated to ensure even distribution of the mixture across the cell layer.

### 2.10 CCK‐8 Assay

Assays were conducted following the protocol provided with the CCK8 kit (Beyotime, Shanghai, China). Human astrocytes were seeded into 96‐well plates at a density of 1 × 10^4^ cells per well, with 100 μL of cell suspension added to each well. After incubation overnight, small molecule inhibitors were added to respective wells as per experimental designs, with at least 3 replicate wells prepared for each treatment condition. The original medium in the 96‐well plate was aspirated, and 100 μL of fresh medium supplemented with small molecule inhibitors was added to each well. The treated cells were further incubated at 37°C in a 5% CO_2_ incubator for 24–72 h. Upon completion of incubation, 10 μL of CCK‐8 reagent was added to each well. The 96‐well plate was gently shaken to ensure thorough mixing, then incubated again at 37°C for 4 h. After this final incubation step, the absorbance (OD value) of each well was determined at 450 nm using a microplate reader.

### 2.11 ELISA Assay

The ELISA kits used in this study were sourced as follows: the kit for detecting C3 was obtained from Wuhan FineTest (Wuhan, China); the kit for S100A10 detection was purchased from JONLNBIO (Shanghai, China); and the kits for assaying IL‐1α, IL‐33, and HMGB1 were all acquired from Beyotime (Shanghai, China). All tests were performed strictly in accordance with the protocols provided with the respective kits. Cell supernatants from each group were collected and centrifuged at 1000 × *g* for 20 min at 4°C, and the supernatant was retained for analysis. A 100 μL aliquot of each sample was dispensed into designated wells of a 96‐well ELISA plate, in duplicate. The plate was sealed and incubated at 37°C for 90 min. After incubation, the liquid was removed, and 100 μL of biotinylated detection antibody working solution was added to each well, followed by incubation at 37°C for 1 h. The plate was then rinsed three times, after which 100 μL of HRP enzyme conjugate working solution was added to each well. Following a 30‐min incubation at 37°C, the wells were rinsed three times again. Next, 90 μL of TMB substrate solution was added to each well; the plate was covered and incubated at 37°C for 15 min. The reaction was halted by adding 50 μL of stop solution to each well, and the absorbance of each well at 450 nm was measured immediately using a microplate reader.

### 2.12 Real‐Time Fluorescence Quantitative Polymerase Chain Reaction (RT‐qPCR) Assay

Total RNA was extracted using TRIzol reagent (Invitrogen, USA), followed by reverse transcription into complementary DNA (cDNA) with the RevertAid First Strand cDNA Synthesis Kit (Thermo, USA). Subsequently, quantitative real‐time PCR (qPCR) analysis was conducted on the obtained cDNA samples using SYBR qPCR Master Mix (Vazyme, Nanjing, China). Glyceraldehyde‐3‐phosphate dehydrogenase (GAPDH) was used as the internal reference gene to normalize the expression levels of target genes, and the relative expression levels of the target genes were determined via the 2‐ΔΔCt method. Primer sequences are listed in Table 1.

**TABLE 1:** RT‐qPCR primer sequence.

Gene	Forward primer (5′→3′)	Reverse primer (5′→3′)
*RIPK1*	AAAGCCCACAGAGAAGTCGG	GTCATCCACATCTGGCCTGT
*RIPK3*	GCAACATAGGAAGTGGGGCT	GGTCCCAGTTCACCTTCTCG
*JAK1*	GCTCCAAATCGCACCATCAC	CAGTGAGCTGGCATCAAGGA
*JAK2*	CCCTTGGGAAATCTGAGGCA	TGGAAGACATGGTTGGGTGG
*JAK3*	ATGAGCCAAGTGTCGTACCG	GTAGGCCAGCTGTTTGACCA
*TYK2*	GCTCAATGTAGTCCCAGCCT	GGAATGATGGCCTCTGGGTT
*STAT1*	ACAAAGTCATGGCTGCTGAGA	GAAGGGTGAACTTCAGACACA
*STAT2*	GCTGCACTTGGGAGTGATGA	AGGATCCTGGGAAAAGGGCT
*STAT3*	GAGCTGCACCTGATCACCTT	GCTTGGGATTGTTGGTCAGC
*STAT4*	ATCGTACGTTGGTCGTGGTC	TCCTTGCAGAACTTGGCCC
*STAT5a*	TATGTGTTTCCTGACCGCCC	CTCAGGGACCACTTGCTTGA
*STAT5b*	AGCTGCAGAAGAAGGCAGAG	AGCGGTCATACGTGTTCTGG
*STAT6*	CTGCTGCAACTTGGCTAGTG	CAAGGGTGCTGATGTGTTGC
*MLKL*	ATCTCTCACCTCAGGAACTGGA	AGAATTACAGCCTTGAAACGGG
*GAPDH*	CAAATTCCATGGCACCGTCA	GACTCCACGACGTACTCAGC

### 2.13 Western Blot

Total protein was extracted and concentrations were determined using a BCA assay kit. A 50 μg aliquot of protein per sample was separated by SDS‐PAGE on a 4%–20% gradient gel and transferred to PVDF membranes. The membranes were blocked with 5% BSA for 1 h, incubated with primary antibodies overnight at 4°C, and then with HRP‐conjugated secondary antibodies for 1 h at room temperature. Protein bands were visualized using ECL reagent, with β‐actin serving as the internal reference. The gray values of target protein bands were quantified using Image J software. The antibodies used in the experiment were sourced as follows: Rabbit anti‐phospho‐RIPK1, rabbit anti‐phospho‐RIPK3, GFAP, PHD2, phospho‐MLKL, SERPING1, FKBP5, AMIGO2, EMP1, S100A10, PTX3, phospho‐STAT1, phospho‐STAT2, phospho‐STAT3, phospho‐STAT4, phospho‐STAT5, phospho‐STAT6, phospho‐JAK1, phospho‐JAK2, phospho‐JAK3, and phospho‐TYK2 monoclonal antibodies were all purchased from Abcam (Cambridge, UK). The mouse monoclonal HIF1α antibody was obtained from Santa Cruz (Shanghai, China). Rabbit anti‐phospho‐VHL and β‐actin polyclonal antibodies were sourced from Affinity Biosciences (Jiangsu, China). Note: During protein extraction, maintain samples on ice to prevent protein degradation; ensure complete transfer of proteins to PVDF membranes by checking the transfer efficiency of pre‐stained markers; and avoid prolonged exposure to ECL reagent to prevent overexposure of bands.

### 2.14 Transmission Electron Microscopy

Human astrocytes were collected by centrifugation, fixed with 2.5% glutaraldehyde for 6 h at 4°C. The cells were rinsed three times with 0.1 M phosphate buffer, 15 min each time. Post‐fixed with 1% osmium tetroxide in the dark for 1 h, they were dehydrated through a graded ethanol series. Infiltration was performed with a 1:1 mixture of epoxy resin and acetone for 2 h, followed by pure resin overnight. The embedding blocks were trimmed into a trapezoidal shape, and sections were cut to a thickness of approximately 70 nm. The sections were then stained with 2% uranyl acetate at room temperature for 30 min, mounted on copper grids, and subjected to carbon coating. The mitochondrial cristae structures in the cells were observed under an electron microscope.

### 2.15 Statistical Analysis

The statistical analysis utilized GraphPad Prism 9.0 software (San Diego, USA). Statistical significance was established at *p* < 0.05. The *p*‐value was calculated using the independent *t*‐test and ANOVA. Each experimental data set comprised three biological replicates.

## 3 Results

### 3.1 Hypoxia and Astrocyte Necroptosis Occur After Cavity and Scar Formation in Spinal Cord‐Injured Rats, and the JAK/STAT Signaling Pathway May Be the Key Pathway Involved

**FIGURE 2:**
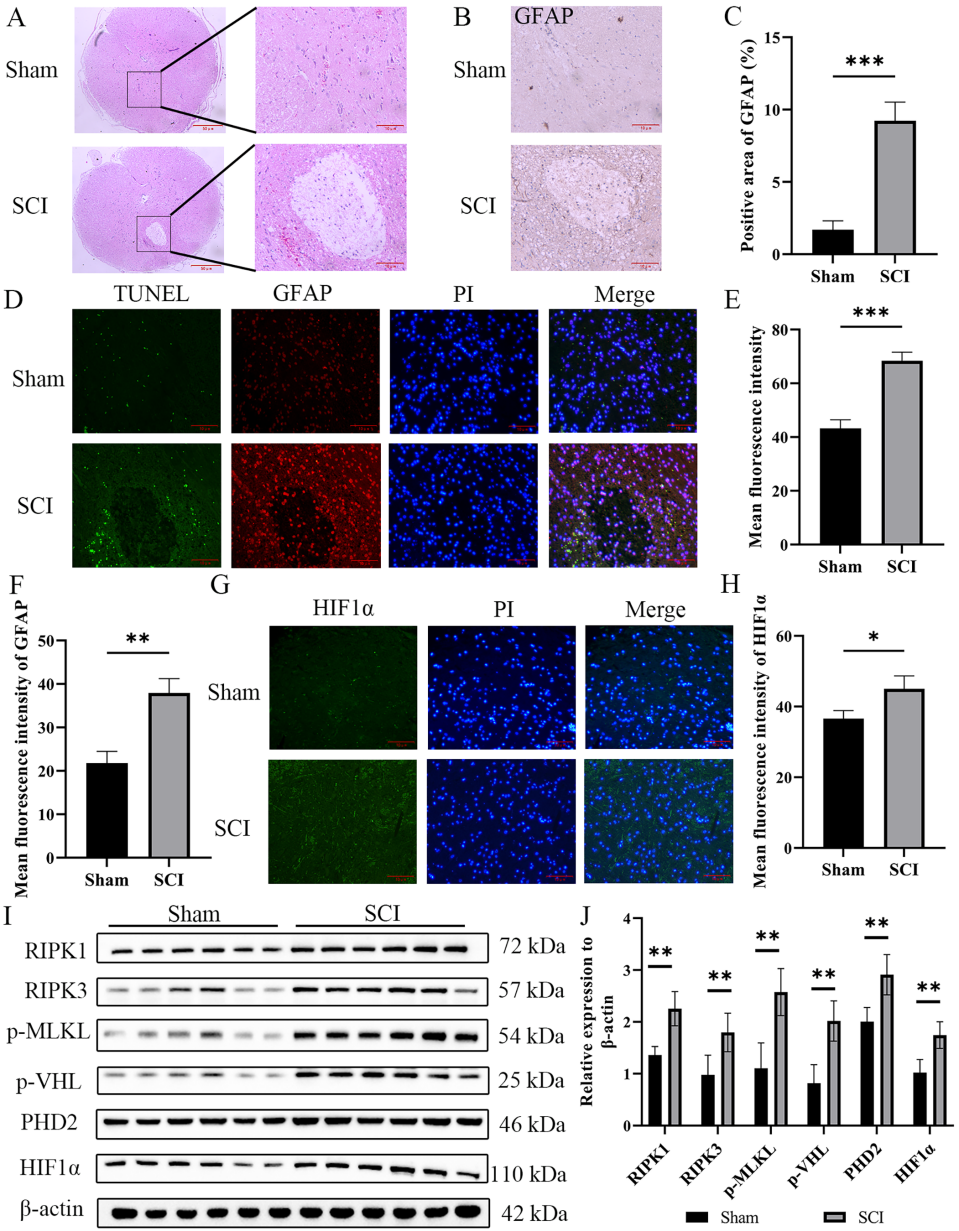
Hypoxia and astrocyte necroptosis occur after cavity and scar formation in spinal cord‐injured rats. (A) HE staining to observe the pathology of the injured area after SCI in rats. (B and C) Immunohistochemical experiments were performed to detect GFAP expression in the injured area after SCI in rats, and the GFAP‐positive area was counted using Image J. (D–F) Immunofluorescence double‐labeling was used to detect GFAP and apoptosis in the area of SCI injury in rats. (G, H) Tissue immunofluorescence detection of HIF1α fluorescence intensity in the region of SCI injury in rats. (I, J) The expression of RIPK1, RIPK3 and the phosphorylation level of MLKL, as well as the levels of p‐VHL, PHD2 and HIF1α, key factors of the HIFα pathway, were detected by Western blotting in the injured tissues after SCI in rats. The blot bands were analyzed in grayscale with Image J software, and β‐Actin was used as an internal reference for relative quantification. *n* = 6; *p < 0.05, **p < 0.01, ***p < 0.001.

To investigate whether hypoxia and astrocyte necroptosis occur after SCI, we established a rat SCI model and assessed pathological status of SCI lesion tissues, as well as the levels of hypoxia and necroptosis markers. HE staining showed that compared with Sham group rats, SCI model rats exhibited cystic cavities in the lesion tissues, with sparse inflammatory cell infiltration around them, disorganized structural arrangement, and glial scar formation (Figure 2A). Immunohistochemical results revealed a significant increase in GFAP expression area around the injury cavity after SCI in rats (Figure 2B,C). Immunofluorescence double staining was used to further detect astrocyte apoptosis in the SCI lesion area, showing that GFAP expression in the injury area was significantly higher in SCI rats than in Sham group rats. Meanwhile, apoptosis was significantly increased in cells with high GFAP expression (Figure 2D–F). Hypoxia‐inducible factor HIF1α expression in the SCI lesion area was detected by tissue immunofluorescence. Results showed that the fluorescence intensity of HIF1α was significantly enhanced in the SCI group compared with the Sham group (Figure 2G,H). Western blot assays revealed significantly upregulated expression of necroptosis proteins RIPK1, RIPK3, and p‐MLKL, as well as key hypoxia‐inducible proteins HIF1α and p‐VHL, in lesional tissues of SCI rats (Figure 2I,J).

**FIGURE 3:**
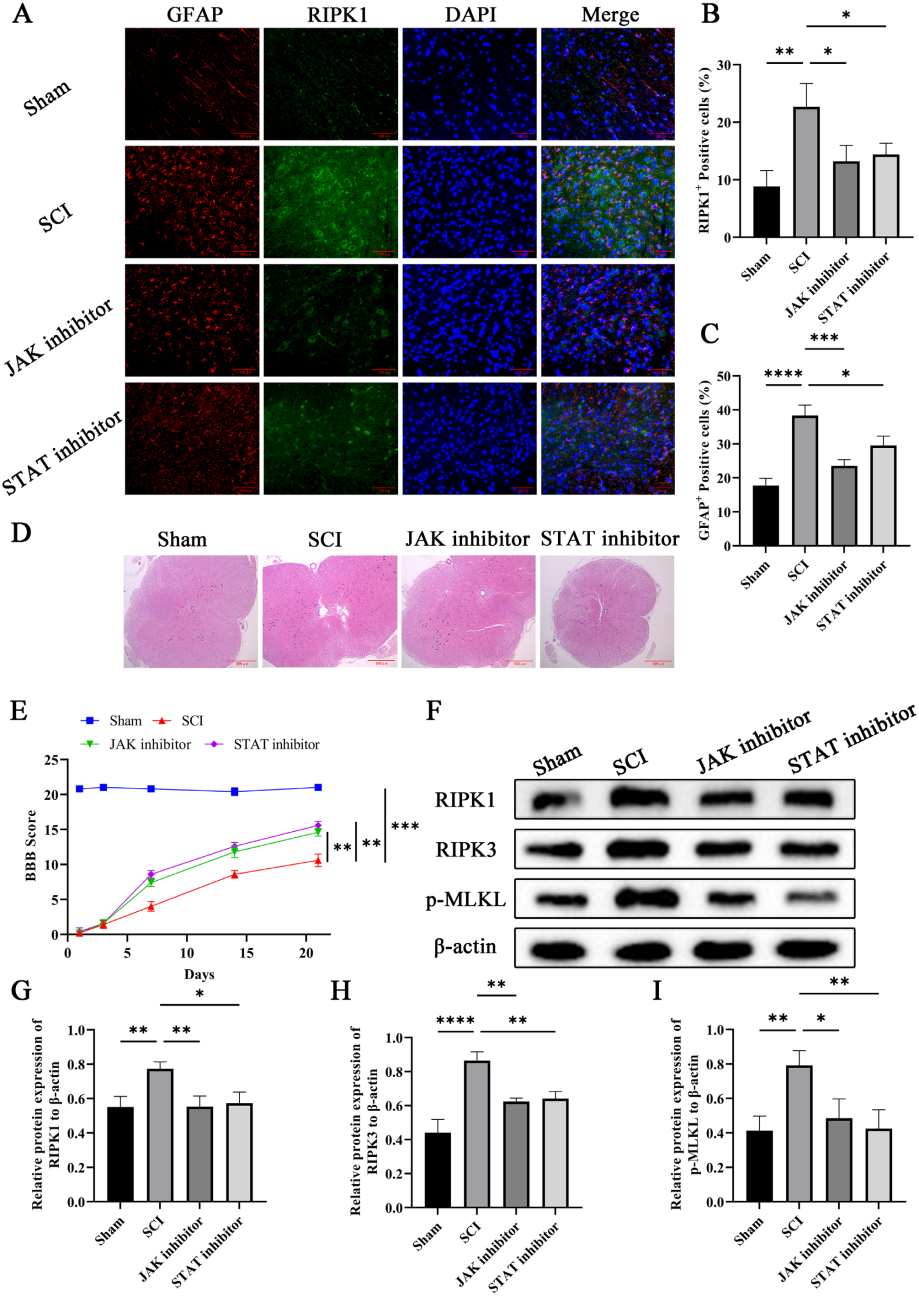
The JAK/STAT signaling pathway may act as a key pathway mediating astrocytic necroptosis. (A–C) Double immunofluorescence double‐labeling was performed to detect astrocytic necroptosis in the injured spinal cord regions of SCI rats (Scale bar = 100 μm), and ImageJ software was used for quantitative analysis of GFAP‐ and RIPK1‐positive areas. (D) Hematoxylin and eosin (H&E) staining was conducted to observe the pathological changes in the injured spinal cord regions of rats following SCI (Scale bar = 500 μm). (E) BBB locomotor scores were evaluated in rats at 1, 3, 7, 14, and 21 days post‐injury (dpi) after SCI. (F–I) Western blot analysis was employed to detect the protein expressions of RIPK1 and RIPK3, as well as the phosphorylation level of MLKL, in the injured spinal cord tissues of rats following SCI. Grayscale analysis of the blot bands was carried out with ImageJ software, and relative quantification was normalized to β‐Actin as the internal reference. *n* = 5; *p < 0.05, **p < 0.01, ***p < 0.001.

Furthermore, double immunofluorescence staining and Western blot assays were performed to detect astrocytic necroptosis in the SCI tissues of SCI rats after JAK–STAT pathway inhibition. The results demonstrated that treatment with JAK or STAT inhibitors significantly suppressed the positive signals of GFAP and RIPK1 in the SCI tissues of SCI model rats and also caused a significant decrease in the protein expressions of RIPK1 and RIPK3 as well as the phosphorylation level of p‐MLKL (Figures 3A–C and 2F–I). We also examined the histopathological status of SCI tissues and conducted BBB locomotor scale assessments in SCI model rats following intervention with JAK or STAT inhibitors so as to confirm the effects of the JAK–STAT pathway on SCI and post‐injury functional recovery. The results showed that compared with the sham‐operated group, SCI model rats had a significant reduction in BBB scores, with a score of 0 at 1 day post‐injury. During the subsequent 21‐day observation period, BBB scores in the SCI model group recovered gradually but remained significantly lower than those in the control group. Notably, SCI model rats receiving JAK or STAT inhibitor intervention exhibited a markedly accelerated recovery of BBB scores relative to the untreated SCI model group. Meanwhile, these functional deficits were accompanied by corresponding alterations in syringomyelia. Following treatment with JAK or STAT inhibitors, a marked reduction in the size of syringomyelia was observed (Figure 3D,E). These findings suggest that the JAK–STAT signaling pathway may promote the progression of SCI by inducing astrocytic necroptosis.

### 3.2 Hypoxia Promotes Astrocyte Necroptosis

**FIGURE 4:**
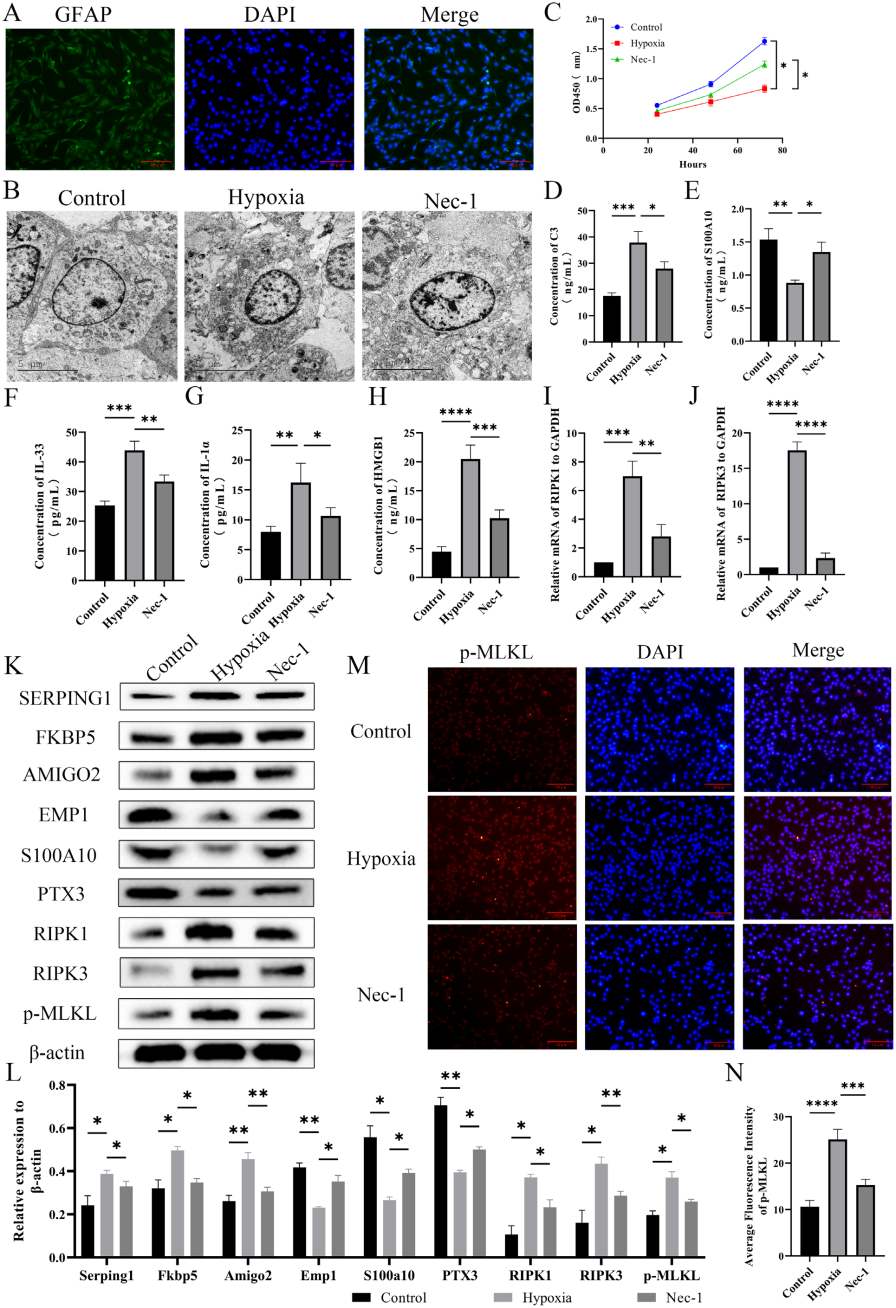
Hypoxia promotes astrocyte necroptosis. (A) Cellular immunofluorescence detection of GAFP expression in human astrocytes. (B) Transmission electron microscopy of human astrocytes morphology. (C) Human astrocytes activity detected by CCK8. (D, E) C3 and S100A10 levels in human astrocytes detected by ELISA. (F–H) IL‐1α, IL‐33 and HMGB1 levels in human astrocytes detected by ELISA. (I, J) mRNA levels of *RIPK1* and *RIPK3* were detected by RT‐qPCR. (K, L) Western blotting experiments were performed to detect astrocyte A1‐type markers: SERPING1, FKBP5, And AMIGO2; A2‐type markers: EMP1, S100A10 And PTX3; necroptotic apoptosis markers: RIPK1, RIPK3 Expression and MLKL phosphorylation levels. The blot bands were analyzed in grayscale by Image J software and relative quantification was performed using β‐Actin as an internal reference. (M, N) Detection of MLKL activity by cellular immunofluorescence and statistics of its average fluorescence intensity. *n* = 3; *p < 0.05, **p < 0.01, ***p < 0.001, ****p < 0.0001.

Given evidence of hypoxia and astrocyte apoptosis in the injured area after rat SCI, we conducted in vitro mechanistic investigations using human astrocytes through a series of biological experiments. To explore the specific mechanisms of necroptosis in human astrocytes under hypoxic conditions, human astrocytes were induced by hypoxia and treated with the necroptosis inhibitor Nec‐1. After identifying astrocytes via immunofluorescence, hypoxic induction was performed, and cell morphology was observed by transmission electron microscopy (TEM). Results showed that the cells highly expressed the astrocytic marker GFAP, confirming that the cells used in this experiment were astrocytes. TEM results revealed that the ultrastructure of cells in the control group was intact, with normal organelle structures. Compared with the control group, cells in the hypoxia group exhibited significant damage, characterized by nuclear pyknosis and organelle swelling; whereas treatment with Nec‐1 significantly alleviated the ultrastructural damage of the cells (Figure 4A,B). CCK8 assay results showed that hypoxic induction significantly reduced human astrocytes proliferation capacity compared to the control group, which was significantly reversed by Nec‐1 treatment (Figure 4C). To determine the astrocyte subtype after hypoxia induction, ELISA assays were used to detect levels of A1/A2 astrocyte activation markers C3 and S100A10. Results showed that C3 levels were significantly increased and S100A10 levels significantly decreased in human astrocytes after hypoxia induction. Levels of necroptosis‐associated cytokines HMGB1, IL‐1α, and IL‐33 also significantly increased after hypoxia induction, and these changes were significantly reversed by Nec‐1 treatment (Figure 4D–H). Additionally, RT‐qPCR results were consistent with these findings (Figure 4I,J). Western blot results showed that, compared to the control group, hypoxia induction significantly upregulated protein expression of A1 astrocyte activation markers (SERPING1, FKBP5, AMIGO2) and downregulated A2 astrocyte activation markers (EMP1, S100A10, PTX3) in human astrocytes. Key necroptosis factors (RIPK1, RIPK3 and MLKL) were also upregulated, and these changes were suppressed by Nec‐1 (Figure 4K–N).

### 3.3 Hypoxia Promotes Astrocyte Necroptosis via the JAK–STAT Pathway

**FIGURE 5:**
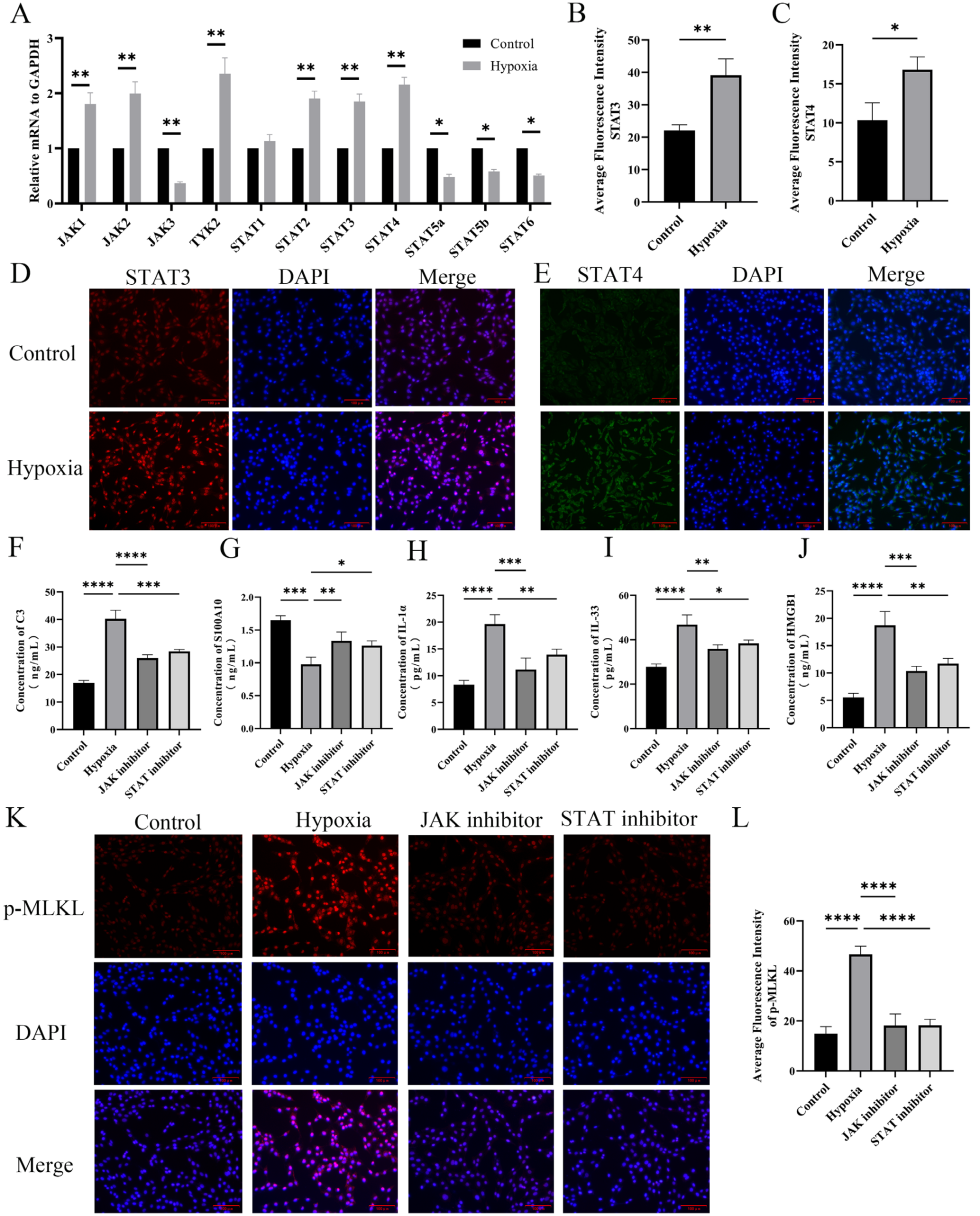
Hypoxia promotes astrocyte necroptosis via the JAK–STAT pathway. (A) *JAK* family: *JAK1‐3*, *TYK*, and *STAT* family: *STAT1‐6* mRNA levels detected by RT‐qPCR. (B–E) Cellular immunofluorescence assay for STAT3 and STAT4 activity, quantification of their mean fluorescence intensity and statistics using image J software. (F, G), C3 and S100A10 levels in human astrocytes detected by ELISA. F and G, IL‐1α, IL‐33 and HMGB1 levels in human astrocytes detected by ELISA. (H–J) mRNA levels of *RIPK1*, *RIPK3*, and *MLKL* were detected by RT‐qPCR. (K, L) Detection of MLKL activity by cellular immunofluorescence and statistics of its average fluorescence intensity. *n* = 3; *p < 0.05, **p < 0.01, ***p < 0.001, ****p < 0.0001.

Studies have shown that STAT3 may be a key regulator of reactive astrocyte proliferation after SCI [21], and its mediated JAK/STAT signaling pathway may play a critical role in regulating astrocyte pathological activities after SCI. To investigate the specific molecular mechanisms of this signaling pathway in human astrocytes under hypoxic conditions, RT‐qPCR was performed to measure the transcriptional levels of major regulatory genes in the JAK/STAT pathway (including *JAK1‐3*, *TYK2*, *STAT1‐6*) (Figure 5A). These genes were significantly upregulated under hypoxia. Further immunofluorescence assays for key JAK/STAT signaling factors STAT3 and STAT4 revealed that both were significantly activated in hypoxia‐induced human astrocytes compared to normoxic human astrocytes, with the effect of STAT3 being evident (Figure 5B–E). These results indicated the important role of the JAK/STAT signaling pathway in hypoxia‐induced astrocytes. Thus, JAK inhibitors and STAT inhibitors were used to treat hypoxia‐induced human astrocytes to further explore whether the JAK/STAT signaling pathway is associated with hypoxia‐induced astrocyte necroptosis. ELISA results showed significantly increased levels of IL‐33, HMGB1, IL‐1α, and C3, and decreased S100A10 levels after hypoxia induction, all of which were significantly reversed by JAK and STAT inhibitor treatment (Figure 5F–J). Meanwhile, hypoxia induction promoted MLKL activation in human astrocytes, which was significantly inhibited by JAK and STAT inhibitor treatment (Figure 5K,L).

### 3.4 Hypoxia Promotes Astrocyte Necroptosis by Activating JAK–STAT Pathway via HIF‐1α

**FIGURE 6:**
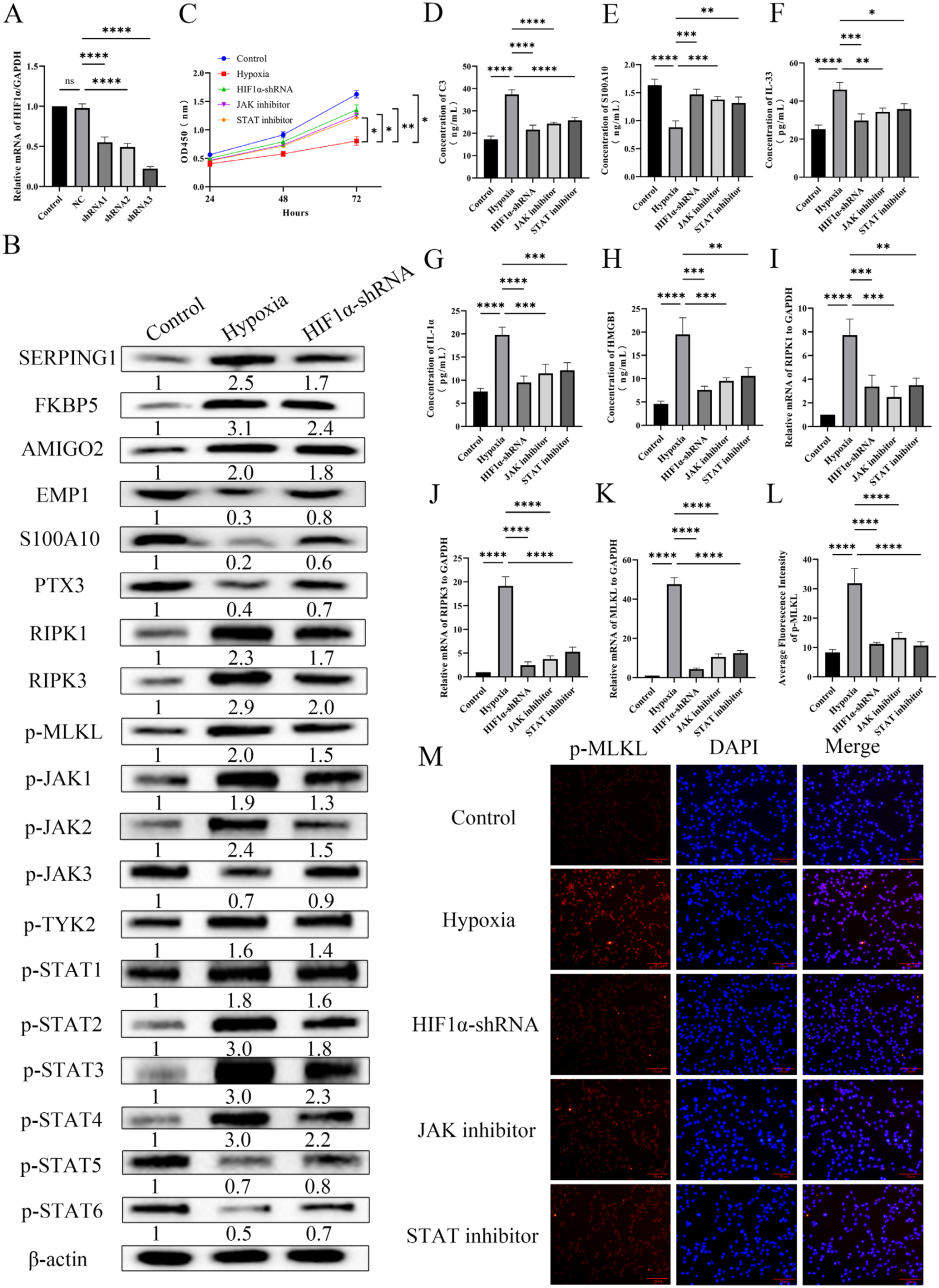
Hypoxia promotes astrocyte necroptosis by activating JAK–STAT pathway via HIF‐1α. (A) *HIF1α* knockdown inefficiency detected by RT‐qPCR assay. (B) Western blot experiments were performed to detect astrocyte type A1 markers: SERPING1, FKBP5, and AMIGO2, type A2 markers: EMP1, S100A10, and PTX3, expression of necroptotic apoptosis key proteins RIPK1, RIPK3, and phosphorylation level of MLKL, and phosphorylation levels of JAK family: JAK1‐3, TYK, and STAT family: STAT1‐6. The blotting bands were analyzed in grayscale with image J software, and relative quantification was performed using β‐Actin as an internal reference. The numbers below the blot bands are the relative expression values for each band. (C) Proliferative activity of human astrocytes in each group was assayed by CCK assay. (D, E) C3 and S100A10 levels in human astrocytes detected by ELISA. (F–H) IL‐1α, IL‐33, and HMGB1 levels in human astrocytes detected by ELISA. (I–K), mRNA levels of *RIPK1*, *RIPK3*, and *MLKL* were detected by RT‐qPCR. (L, M), Detection of MLKL activity by cellular immunofluorescence and statistics of its average fluorescence intensity. *n* = 3; *p < 0.05, **p < 0.01, ***p < 0.001, ****p < 0.0001.

HIF1α regulates cellular metabolism and survival in hypoxic environments, with studies indicating its critical role in SCI [22]. HIF1α‐knockdown was established in human astrocytes using shRNA. RT‐qPCR confirmed that all three tested shRNAs significantly reduced HIF1α mRNA, with shRNA3 showing the highest knockdown efficiency and therefore selected for further experiments (Figure 6A). Western blot assays revealed that HIF1α knockdown significantly inhibited hypoxia‐induced upregulation of SERPING1, FKBP5 and AMIGO2, as well as downregulation of EMP1, S100A10 and PTX3. Additionally, hypoxia induction increased RIPK1 and RIPK3 expression and activated MLKL, while significantly activated JAK1‐3, TYK2, and STAT1‐6—all of which were reversed by HIF1α knockdown (Figure 6B). CCK8 results showed that hypoxia induction significantly reduced cell viability, which was partially restored by either HIF1α expression inhibition or JAK/STAT signaling pathway inhibition (Figure 6C). ELISA results demonstrated significantly increased C3, IL‐33, HMGB1, and IL‐1α levels, and decreased S100A10 levels after hypoxia induction, all of which were significantly reversed by HIF1α knockdown or JAK/STAT inhibitor treatment (Figure 6D–H). Furthermore, RT‐qPCR and immunofluorescence assays showed that inhibition of HIF1α expression and the JAK/STAT signaling pathway significantly reversed RIPK1 and RIPK3 transcriptional levels and MLKL activation (Figure 6I–M). Collectively, these results indicate that hypoxia induction promotes astrocyte differentiation toward the A1 phenotype and induces necroptosis in A1‐type astrocytes via the HIF1α‐mediated JAK/STAT signaling pathway.

### 3.5 Hypoxia Regulates RIP1 Expression via Activated JAK–STAT Pathway to Promote Astrocyte Necroptosis

**FIGURE 7:**
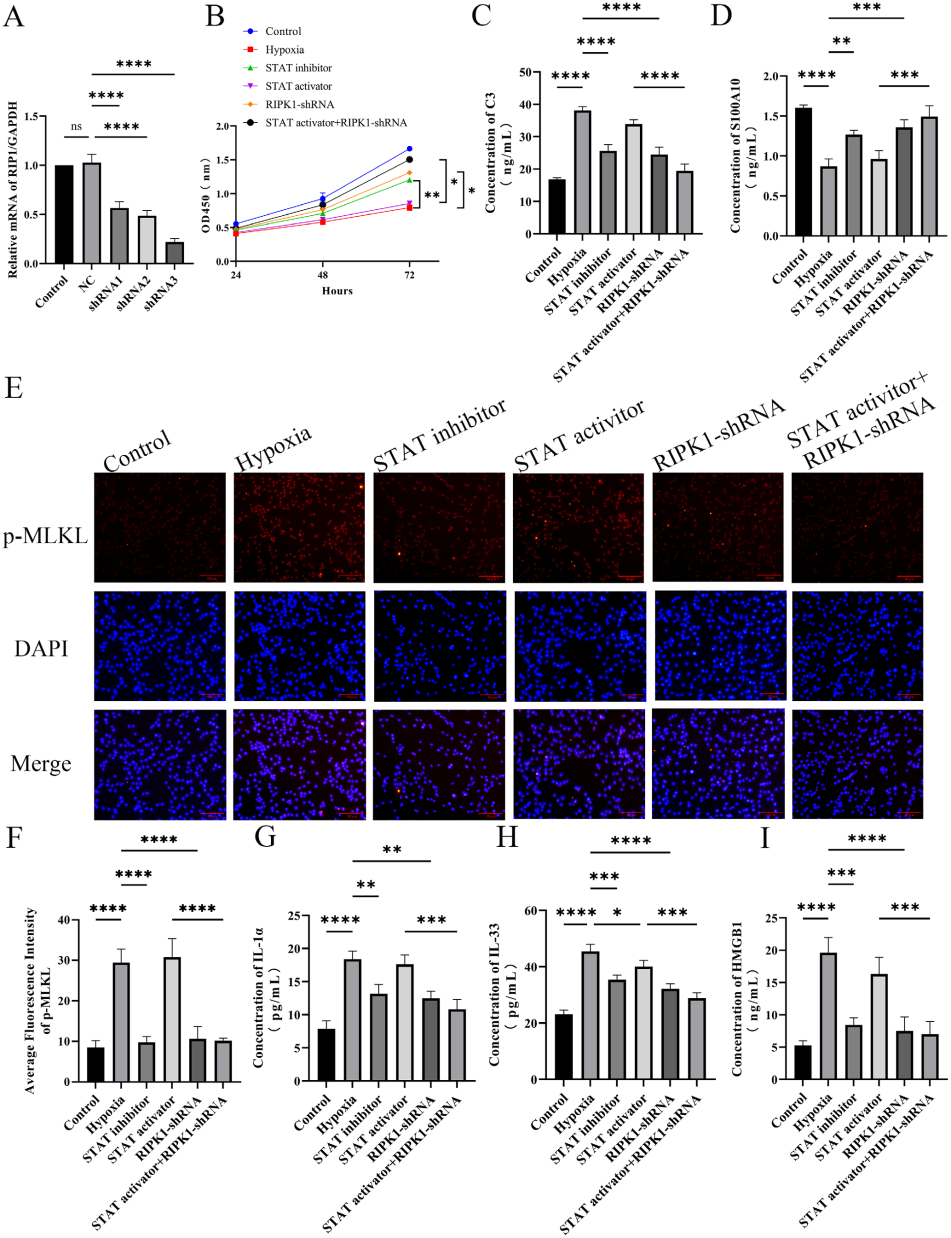
Hypoxia regulates RIP1 expression via activated JAK–STAT pathway to promote astrocyte necroptosis. (A) *RIP1* knockdown inefficiency detected by RT‐qPCR assay. (B) Human astrocyte proliferation viability was assayed by CCK8 assay, shown as OD values at OD450 nm. (C, D) C3 and S100A10 levels in human astrocytes detected by ELISA. (E, F) The level of MLKL phosphorylation in human astrocytes was detected by cellular immunofluorescence, and the fluorescence intensity was analyzed and statistically analyzed by Image J software. (G–I) IL‐1α, IL‐33 and HMGB1 levels in human astrocytes detected by ELISA. *n* = 3; *p < 0.05, **p < 0.01, ***p < 0.001, ****p < 0.0001.

The necroptosome is a protein complex that regulates necroptotic cell death, with RIPK1 being a major component. To further validate the molecular mechanism by which hypoxia induction promotes A1 astrocyte necroptosis via the HIF1α‐JAK/STAT pathway, knocked down RIPK1 using shRNA and modulated STAT signaling with an activator or inhibitor. RT‐qPCR confirmed efficient *RIPK1* knockdown, and results showed successful *RIP1* knockdown; the shRNA with the highest knockdown efficiency was selected for transfection into human astrocytes to establish RIPK1‐knockdown cell lines (Figure 7A). CCK8 results showed significantly decreased cell viability in the hypoxia‐induced group compared to the control group, which was significantly attenuated by RIPK1 knockdown or STAT inhibitor treatment. STAT activator had no obvious effect on hypoxia‐induced cell viability, but RIP1 knockdown in this context maximally restored human astrocytes' viability (Figure 7B). Additionally, hypoxia induction significantly promoted C3 elevation and S100A10 reduction (Figure 7C,D). Immunofluorescence revealed that p‐MLKL activation was suppressed by RIPK1 knockdown or STAT inhibition under hypoxia. Compared with hypoxia plus STAT activator alone, the addition of RIPK1 knockdown further enhanced p‐MLKL fluorescence intensity (Figure 7E,F). Meanwhile, ELISA results showed consistent trends in IL‐33, HMGB1, and IL‐1α levels (Figure 7G,H).

## 4 Discussion

Regenerative repair of SCI has long been a major clinical challenge in the treatment of SCI, with effective therapies still lacking. SCI repair depends on the plasticity of neural circuits. However, the complexity of the pathological processes in SCI makes the remodeling of neural circuits particularly difficult [23]. A comprehensive and in‐depth exploration of the pathological mechanisms during SCI progression can provide more key intervention targets for both neural circuit remodeling and SCI repair. In this study, we investigated the specific molecular mechanisms occurring in astrocytes after SCI, demonstrating that hypoxia, COI expansion, and astrocyte cell death occur in the lesion area following SCI. In vitro experiments confirmed that these phenomena might be mediated by necroptosis promoted by the activation of the HIF1α‐JAK–STAT signaling pathway.

Astrocytes are present throughout the spinal cord and play a crucial role in the CNS under both physiologic and pathologic conditions, including assisting in the formation of neural circuits. In the early stages of SCI, astrocytes proliferate extensively and undergo marked morphological polarization, characterized by the lengthening, thickening, and directional extension of protrusions. This polarization is critical in the filling of spinal cord COIs and the establishment of a suitable local environment for axonal regeneration. Therefore, elucidating the molecular mechanisms of astrocyte pathology after SCI is essential. Reactive astrocytes have a two‐fold role in SCI repair. On the one hand, as the main constituent cells of glial scars, they are activated after SCI to encapsulate the injury site. This helps limit the expansion of the inflammatory response and maintain the integrity of surrounding cells. Thus, reactive astrocytes are vital for sealing the injury in the early phase. On the other hand, in later stages, they release inflammatory factors that damage nerve cells and impede nerve repair in the late stage of SCI by establishing a physicochemical barrier [7, 24, 25, 26]. These phenomena are likely linked to astrocyte phenotypic differentiation. There are two main subtypes of reactive astrocytes, the neurotoxic A1‐type and the neuroprotective A2‐type. Astrocytes can be driven toward the A1 phenotype through the NF‐κB signaling pathway and to the A2 phenotype through the STAT3 signaling pathway [27, 28]. Studies indicate that reactive astrocytes are activated at the site of injury after SCI, and that knockdown of Celsr2 results in changes in astrocyte morphology and phenotype, including reduced COI and glial scar area, promoting nerve fiber regeneration and enhancing neurological function recovery [9]. This study emphasized the important role of reactive glial cell phenotypic changes in post‐SCI repair in mice. In our study, assessment of GFAP expression level in the injured tissues after SCI in rats showed that reactive astrocytes in the injured area after SCI showed significant proliferation, which may be related to their phenotypic transformation and the formation of glial scar around the COI.

Maintenance of organismal homeostasis is closely tied to oxygen concentration in the body, and prolonged exposure to hypoxic stress may induce neural injury. Cells activate the HIFα pathway during short‐term hypoxia to adapt to low‐oxygen environments. Upon sustained hypoxia, RIP1 is activated, triggering downstream necroptosis [20]. Necroptosis of astrocytes is highly detrimental to neural repair after SCI, as it induces cellular oxidative stress and releases pro‐inflammatory signals, exacerbating SSCI and contributing to further neurological dysfunction and impaired repair [29]. Consistent with these findings, animal experiments in this study showed that SCI resulted in COI and hypoxic conditions in the injured tissue. In the hypoxic microenvironment, the HIFα pathway and key necroptosis factors were significantly activated in tissues surrounding the COI at the injury site, alongside increased necroptosis of reactive astrocytes. Studies have demonstrated that COI presence disrupts the normal spinal cord architecture and neural signal transmission, creating a microenvironment that hinders neural regeneration [30]. Therefore, the hypoxic microenvironment in the injured area of rats after SCI may drive astrocyte polarization toward the A1 phenotype, intensify inflammatory responses, eliminate cells via necroptosis, promote gradual expansion of COI area, and ultimately accelerate SCI progression.

To validate whether astrocytes undergo necroptosis under hypoxic conditions after SCI and the specific molecular mechanisms, hypoxic induction experiments were performed on human astrocytes with knockdown of HIF1α or RIP1. Results showed that hypoxia induced phenotypic transformation of astrocytes toward the A1 type, upregulated HIF1α expression, and increased levels of key necroptosis factors. Treatment with necroptosis inhibitors or knockdown of HIF1α/RIP1 reversed the cellular effects of hypoxia on human astrocytes. These findings further confirm that hypoxia regulates astrocyte phenotypic transition and promotes necroptosis through the HIF1α signaling pathway.

The activation of JAK and the phosphorylation of its downstream transcriptional signal STAT, a cascade signaling pathway, play a critical role in the pathological progression after SCI. The JAK/STAT signaling influences cell growth, survival, and differentiation. The JAK family includes JAK1, JAK2, JAK3, and TYK2; the STAT family consists of STAT1, STAT2, STAT3, STAT4, STAT5A, STAT5B, and STAT6 [31]. Reports have shown that STAT3 is activated in reactive astrocytes within the injured area after SCI [22]. Furthermore, oxidative stress activates the JAK/STAT1 pathway in the basilar artery of the subarachnoid space, which may be associated with apoptosis after SCI [31]. Recent studies have demonstrated that the JAK/STAT signaling pathway is aberrantly activated during the progression of SCI, inducing cellular inflammation and necroptosis to exacerbate neuronal injury. During the acute phase of mechanical injury or ischemia–reperfusion, JAK2/STAT3 undergoes phosphorylation and triggers the release of downstream proinflammatory cytokines (IL‐6, TNF‐α), while synergizing with the NF‐κB pathway to amplify the inflammatory response [32], thus forming a neurotoxic microenvironment. Sustained activation of STAT3 at this stage not only promotes the aberrant proliferation of astrocytes but also enhances cellular necroptosis by upregulating the RIPK1/3 signaling axis [33], ultimately leading to programmed necrosis of neurons. To date, there are relatively few reported investigations into the mechanisms underlying the regulation of RIPK1/3—key proteins mediating cellular necroptosis—by the STAT3 signaling pathway after SCI. In the present study, hypoxic‐induced astrocytes were treated with a JAK inhibitor as well as STAT activators and inhibitors. Our findings revealed that inhibition of the JAK/STAT signaling pathway reversed hypoxic‐induced cellular phenotypic transformation, RIP1 expression, and necroptosis.

In short, this study demonstrates via in vitro and in vivo experiments that activation of the HIF‐1α–JAK–STAT pathway under hypoxic conditions promotes the formation of the COI after SCI, which in turn triggers necroptosis in astrocytes.

## Author Contributions

**Hangchuan Bi** and **Wan Zhang:** conceptualization, methodology, resources, writing – original draft preparation, substantial contributions to the design of the work. **Hao Duan**, **Chao Wang**, and **Xianglin Shen:** methodology, validation, software, the acquisition and analysis of data for the work. **Gang Jiang**, **Haiyan Xue**, **Rongji Yan**, and **Yuan Xu:** visualization, formal analysis, methodology, revising the article critically for important intellectual content. **Yihe Zhang** and **Haoyu Zhao:** validation, software, revising the article critically for important intellectual content. **Fei He** and **Zhihua Wang:** conceptualization, project administration, resources, supervision, revising the article critically for important intellectual content. All authors have approved the version to be published.

## Funding

This study was supported by Priority Union Foundation of Yunnan Provincial Science and Technology Department and Kunming Medical University (Grant No. 202401AY070001‐353) and (Grant No. 202201AY070001‐085); Clinical Medical Research Center of Radiology and Therapy of Yunnan Province (Grant No. 202505AJ310002); Yunnan Clinical Center for Emergency Traumatic Diseases (Grant No. YWLCYXZX2023300075) and (Grant No. 2024YNLCYXZX0082).

## Ethics Statement

All animal experimental protocols involved in this study were reviewed and approved by the Animal Experiment Ethical Review Committee of Kunming Medical University (Approval Number: kmmu20240627).

## Conflicts of Interest

The authors declare no conflicts of interest.

## Data Availability

The data that support the findings of this study are available on request from the corresponding author. The data are not publicly available due to privacy or ethical restrictions.
